# Social justice for adults with high body weight: a systematic review

**DOI:** 10.1186/s12939-026-02792-4

**Published:** 2026-02-21

**Authors:** Imogen Sophia Weidinger, Leonie Josefa Renelt, Solveig Lena Hansen

**Affiliations:** Institute of Public Health and Nursing Research (IPP), University of Bremen, Universitätsallee 1B, 28359 Bremen, Germany

**Keywords:** Obesity, Policy, Prevention, Responsibility, Social Justice, Stigma

## Abstract

**Background:**

Health inequalities that arise form structural and social factors beyond an individual’s control (e.g., socioeconomic status (SES), education access, or neighbourhood and built environment) are widely regarded as unjust in public health ethics. However, to be translated into practice, meaningful calls for greater social justice in health must be grounded in a clear and robust conception of justice. Against this backdrop, this systematic review examines how notions of social justice are conceptualized and operationalized in responses to public health challenges, using obesity as paradigmatic example.

**Methods:**

Following a PRISMA guideline for systematic reviews on ethics literature we searched PubMed, Web of Science, and BeLit in March 2024 and identified 1,377 titles. Screening, data extraction, and analysis were conducted independently by two authors. A total of 33 texts were included in this study. The analysis followed the Qualitative Analysis Guide of Leuven (QUAGOL) framework, providing a structured and theory-oriented interpretation. The qualitative software ATLAS.ti supported the analysis throughout. This systematic review was not registered.

**Results:**

While global inequalities are extensively studied, ethical debates focus mainly on the Global North. The included literature discusses obesity primarily in terms of responsibility, autonomy, disease concepts, stigma, healthcare and food access, as well as prevention. Social justice theories helped to identify injustices and guide policy, although a unified understanding remains emergent.

**Conclusion:**

This paper examines how ethical theories can guide efforts to advance social justice for adults with high body weight (AHBW) and identifies practical steps forward, thereby providing a robust starting point for interdisciplinary research and translational work.

**Supplementary Information:**

The online version contains supplementary material available at 10.1186/s12939-026-02792-4.

## Background

Obesity^1^ has many causes. At the individual level, genetics, mental health, lifestyle choices, and medical factors, in addition to health literacy and self-efficacy, are at play. On a systemic level, obesogenic environments, particularly global and local conditions such as food systems and urban design, influence the accessibility of food, parks, and gyms, as well as access to preventive measures or health care professionals [1]. Global obesity rates are also influenced by social determinants of health (SDH) and drivers of social inequality, such as socio-economic status (SES) [2, 3]. For instance, European data indicates that individuals with lower levels of education have substantially higher rates of obesity, 1.6 times higher among men and 1.9 times higher among women, compared to those with higher education [4]. Furthermore, residing in a neighbourhood with low SES, as opposed to one with high SES, is associated with 31% higher odds of overweight and 45% higher odds of obesity [5].

Whilst health inequalities refer to differences in health that are associated with social advantages or disadvantages, often resulting in worse health for marginalized or socially excluded groups [6], health inequities are “[health] differences which are unnecessary and avoidable but, on addition, are considered unjust” [7] (p. 433). In this context, health equity refers to the systematic reduction of inequalities deemed unjust – for example, by compensating for unequal opportunities or starting conditions – with a specific focus on improving health-related outcomes. What exactly constitute ‘justice’ in this sense, however, is rarely made explicit, leaving understandings of health equity to rest on implicit and often unexamined moral assumptions [8]. Social justice, on the other hand, comprises multiple dimensions that extend beyond the health sector. It addresses the underlying social, economic, and political inequalities that produce disadvantages [9]. Therefore, social justice is often considered the foundation of public health [10, 11] and one of “the fundamental conditions and resources for health” [12] (p. iii).

In practice, the diverse and often competing understandings of justice make it difficult for policymakers and health professionals to determine what is unjust and who bears responsibility for addressing health injustices. Therefore, a robust and well-developed conception of social justice is needed [9, 13]. Without such conceptual clarity, conflicting interpretations are likely to persist, disagreements may be concealed, and policy deliberation can become confused or stalled. This conceptual underdevelopment increases the risk that the ideals of social justice will not be realized, potentially resulting in policies that are muddled, imprecise, or even counterproductive. Luckily there is an array of theoretical frameworks that could clarify what social justice requires in public health [9, 13]. 

Beauchamp and Childress identify four traditional theories of justice, namely, utilitarianism, libertarianism, communitarianism, and egalitarianism, each operating according to different “material principles of distributive justice” [14] (p. 271). These principles help to reflectively distribute resources, particularly in clinical settings. They also highlight the importance of procedural principles when such material principles are unclear, conflicting, or insufficient. Procedural mechanisms (like standardized eligibility procedures, waiting lists, or even lotteries) help guide decision-making processes in situations where no adequate material principle can determine access to scarce medial resources [14]. In line with coherentist approaches^2^, Beauchamp and Childress maintain that: “no single theory of justice or system of distributing health care is solely sufficient for constructive reflection on health policy” [14] (p. 313).

While no single theory of justice fully addresses the complexities of health, systematic engagement with diverse theories of social justice is essential for developing practical ethical tools to tackle health injustices, assign responsibility, and inform policy decisions. On that note, Beauchamp and Childress already presented two theories concerning the values and practices of public health: the *well-being approach* and the *capabilities approach* [14] (pp.270/271)^3^. Additionally, theories such as Nancy Fraser’s scales of justice offer a multi-dimensional framework that highlights how redistribution, recognition, and representation operate across multiple social and political scales, reinforcing the broader point that withing public health ethics, social justice considerations must extend from distributive matters to just relations, discourses, communication, and structures [11].

Against this theoretical background, this paper systematically analyses the ethical discourse on obesity, with a particular focus on issues of social justice. We further examine how notions and theories of justice are applied in this context. Finally, we synthesize the results to develop our own conceptualization of social justice for adults with obesity and discuss how different theories of justice can be operationalized to deepen the understanding of social justice in public health.

We use obesity (medically defined by a BMI ≥ 30 [16]) as a paradigmatic case for examining notions of justice in public health ethics since its affects nearly all medical and care professions. Obesity is not only associated with a wide array of health risks^4^, but also sits at the centre of contested moral, social, and political debates: it involves a broad range of social and structural determinants (e.g., SES and food environments), yet it is simultaneously framed and communicated in public health messaging, clinical discourse, and media representations as a matter of individual choice and responsibility [18, 19]. Additionally, adults with obesity face pervasive stigma in healthcare, employment, and everyday life [20]. The intersection of medical relevance, social disadvantage, and moral judgement makes obesity uniquely suitable for analysing which concepts of justice are invoked, challenged, or overlooked in public health ethics.

## Methods

We decided to conduct a systematic review, a method which has gained significant recognition for its use in bioethics. Some critics deem the use of systematic reviews unfit for ethical debates, arguing that the value-laden nature of ethical reasoning precludes objective summary and risks conflating personal views with scientific analysis [21]. Conversely, others argue that a systematic review of ethical literature aims to analyse concepts, norms, and their practical implications [22]. Furthermore, systematic reviews support research and decision-making by consolidating all pertinent literature [23]. By applying a structured search, selection, and analysis process, they ensure that all relevant perspectives are captured, patterns, conflicts, and gaps are identified, and provide a rigorous foundation for ethical reasoning and policy guidance. To conduct a systematic review, adherence to reporting guidelines is recommended. The Preferred Reporting Items for Systematic Reviews and Meta-Analyses (PRISMA) is recognized internationally for this purpose, but it is only partially suitable for ethical arguments, norms, principles, or conclusions. Therefore, we adopted an approach that further developed PRISMA guidelines for systematic reviews in the field of applied ethics [24]. This gave us a comprehensive framework and a systematic theoretical guide to the specific steps.

### Inclusion criteria

We included texts if the following conditions were met:

1. Language: English.
2. Population: Adult people with obesity.
3. In addition to the well-established medical term ‘obesity’, we also used the term ‘fat’, which is mostly used in activism [25].
4. We decided to exclude children for a number of reasons: (a) there is an array of literature on ethical issues regarding obesity prevention in children [26]; (b) children are considered less blameworthy, meaning that ethical aspects of autonomy and responsibility are discussed in a completely different light; (c) while many interventions target children, efficient and ethically sound programs for adults are rarely established; and (d) the socioeconomic position of the family has a significant impact on children [27].
5. Types of publications: Papers, books, book chapters, PhD theses.
6. Ethical considerations are identified in situations of normative uncertainty or disagreement. Such situations often involve conflicts between moral duties, values, or principles, necessitating moral reasoning [28, 29]. The included texts frame, discuss, or address normative issues related to obesity.​.

We set no timeframe. The search was conducted in March 2024, and we included publications published between 2008 and 2021.

### Search strategy and data sources

This systematic literature search was performed using PubMed, Web of Science and BeLIT databases. While PubMed provides medical and biomedical research findings, Web of Science covers multidisciplinary research. BeLIT is a database specifically for ethical literature.

After selecting the databases, we created two search strings (see Table 1) with the help of an informed specialist. The first search string consists of the two search components ‘Justice’ and ‘Obesity’. The second search string consists of three components: ‘Justice,’ ‘Obesity,’ and ‘Ethics’. This was necessary because some publications (which we could only find through the first search string) contained a normative discussion but were not indexed through an ethical keyword. The PubMed and Web of Science databases were screened with two search strings. For BeLIT, we only used the first search string in a slight adaptation. We used the following terms: obes* OR Obesity OR Fat AND Justice OR Social justice OR Health Inequit* AND Ethic* OR Ethic OR Moral. The database search was conducted in March 2024. This systematic review was not registered.

**Table 1:** Search strings employed in this systematic review

**First Search String in Pubmed and Web of Science without Ethics**	
Justice	(justice[tiab] OR justice[MeSH] OR “Health Inequit*“[tiab]) AND
Obesity	(obes*[tiab] OR fat[tiab] OR obesity[MeSH])
**Second Search String in PubMed and Web of Science with Ethics**	
Justice	(justice[tiab] OR justice[MeSH] OR “Health Inequit*“[tiab]) AND
Obesity	(obes*[tiab] OR fat[tiab] OR obesity[MeSH]) AND
Ethics	(ethic*[tiab] OR moral*[tiab] OR ethics[MeSH] OR morals[MeSH])
**Adpatation: First Seach String in BeLIT with Ethics**	
Justice	(justice OR inequity OR equity) AND
Obesity	(obes* OR fat*) AND
Ethics	(ethic* OR moral*)

We developed a data extraction sheet with the following criteria: year of publication, authors, title, authors’ disciplinary background, authors’ origin, addressed moral issues related to obesity, country/health system, theoretical approach, and implications for practice and implications for policy. We used this data extraction sheet throughout the analysis.

Following the steps of QUAGOL [30], the selected texts were read and reread to obtain a holistic understanding of the literature. In the second step, detailed summaries of the publications were written. From these summaries, key concepts and themes were identified to highlight patterns, overlaps, and contrasts, which were then integrated into a broader global scheme. Finally, the conceptual schemes were synthesized into a systematic summary of the results. The qualitative software ATLAS.ti supported the analysis throughout. While less widely known than approaches such as Braun and Clarke’s [31] thematic analysis or conventional content analysis [32], we decided to use QUAGOL as an interpretive qualitative method which emphasizes iterative, concept-driven analysis rather than rigid categorisation. Its structured yet flexible process enhances transparency, accessibility, and methodological rigor in synthesising normative reasoning.

#### Quality appraisal

We used the academic peer review process for quality assessment. If an article was published in an international peer-reviewed journal, it met the quality standard for inclusion in the review. All 33 publications included met this criterion, and no additional quality assessments were conducted within that group.

#### Bias assessment

As this systematic review examines normative literature rather than empirical studies, standard risk-of-bias assessment tools were not used, as they are designed to evaluate methodological bias in empirical research.

## Results

We structure the presentation of our results as follows. Firstly, we describe our study selection process, explaining how 33 texts were included in the analysis. We then provide a brief overview of the overall sample before examining in depth the moral issues addressed in relation to obesity, including autonomy and responsibility, obesity as a disease, stigma, access to healthcare, prevention and food policy, and health as the ‘ultimate’ goal. Our subsequent analysis focuses on how notions of justice were operationalized in the included texts, highlighting how certain lines of argumentation may potentially reinforce injustice and how theories of justice were applied.

### Study selection

**Fig. 1:**
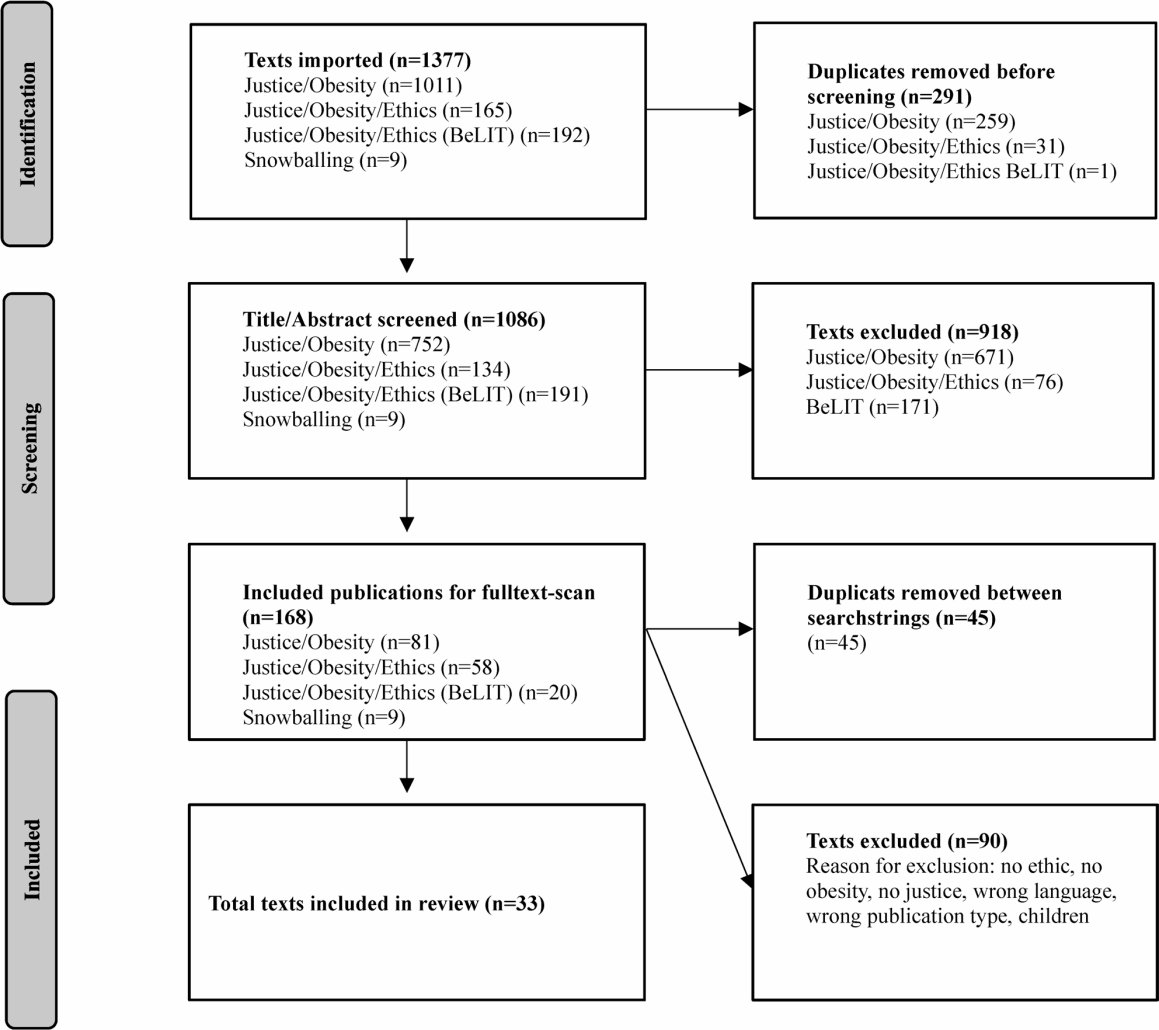
Flow diagram showing the screening process used in this systematic review [33]

We extracted 1,377 titles and abstracts from the databases. After removing duplicates, 1086 titles and their respective abstracts were read independently by reviewer one (first author) and reviewer two (second author). During this stage, 918 texts were excluded for not meeting the inclusion criteria. In the next step, the first and second authors independently reviewed all full texts (168 in total). In addition, if articles referred to a text that was absent from our results but seemed particularly suitable to our topic, they were manually added (snowballing) to the screening process. This led us to include nine additional texts in the full-text screening. Throughout both the abstract and full-text-screening process, disagreement was discussed between the first and second authors. If consensus could not be reached, the last author made the final decision. The last author also read all full texts, which led us to ultimately including 33 text that met the inclusion and exclusion criteria in the systematic review (see Fig. 1 and appendix).

### A brief summary of the overall sample

In the entire sample (1,377 publications), the geographical areas were widely spread. These findings demonstrate that obesity disparity is globally seen as a health problem. Many empirical studies have focused on health inequality, claiming more research is needed to reach health equity.^5^ However, these studies neither described how health equity might be achieved nor elaborated on any moral or political implications of their claims. As a result, when we narrowed our search down to texts with a solid ethical analysis, the few texts that delved deeply into the ethical implications were all from the Global North. 18 of the included texts discussed ethical issues with no particular national context. If a country or healthcare system was referred to, it was mostly the US system (*n* = 12). Other countries/healthcare systems included Canada (*n* = 2), Finland (*n* = 1), Iran (*n* = 1), Australia/New Zealand (*n* = 1) and the UK (*n* = 1). In our analysis below, we acknowledged that different national healthcare systems contain varying prerequisites, roles, and responsibilities.

### Addressed moral issues related to obesity

#### Autonomy and responsibility

The topic of individual responsibility permeated the texts. Authors deliberated over whether individuals make autonomous dietary and lifestyle choices and, if so, whether these choices lead to obesity. For authors who consider dietary and lifestyle choices to extend beyond individuals, it is unjust to blame adults with obesity for their weight and necessary to shift responsibility. For example, Kass et al. argue that governments have a moral duty, grounded in principles of social justice, to foster health-promoting conditions in every sector they oversee or finance [36].

Nielsen and Andersen discuss the concept of backward and forward responsibility for becoming obese. Both types of responsibility were claimed to be insufficient. The multifactorial cause of obesity makes backward-looking responsibility (control over choices and causes of the current situation) impossible. Forward-looking responsibility (holding someone responsible for the expected outcome) is also not sufficient because of the consequences of causing stigma, stress, lower opportunities, and less welfare [37]. 

Sharkey and Gillam proposed three reasons why adults with obesity should not be held responsible for their weight: (1) inability to control unhealthy behaviour, (2) other uncontrollable risk factors for ill health, and (3) personal value of risk-taking [38]. Further Saarni et al. argue that, regardless of cause or responsibility, healthcare should prioritize patient benefit, as health is a basic need and its unequal distribution is ethically problematic. Also, there are many conditions where individuals are responsible for taking risks but are routinely treated in cases of medical need (e.g., extreme sports) [39]. 

#### Obesity as a disease

The question of whether obesity should be considered a disease was discussed frequently. Authors argued, that on the one hand, recognizing a condition as a disease often creates clear duties to offer medical treatment and support. As disease-based discrimination is usually not accepted, such recognition may reduce stigma and improve access to care, thereby potentially improving the circumstances of adults with obesity. Furthermore, framing obesity as a disease could help provide more resources for prevention, treatment, and research, motivating healthcare professionals to perform meaningful and respectable work [40]. However, such medicalization classifies all adults with obesity as sick, including profound societal consequences. It expects that individuals need a cure from their disease [39]. Thus, therapies such as bariatric surgery or weight loss drugs could also put social and moral pressure on adults with obesity [40].

McPhail and Orsini, drawing on the work of the National Association to Advance Fat Acceptance (NAAFA), argue that framing obesity as a disease is deeply entangled with systems of oppression such as racism, ableism, and colonialism. It has been widely documented that marginalized groups have historically been labelled as diseased, reflecting a long-standing pattern of associating pathology with bodies of black, indigenous, and people of colour (BIPOC). The medicalization of fat bodies should therefore be viewed in the context of colonialism, as well as the historical neglect and inadequate care provided to members of BIPOC communities [41]. 

#### Stigma

Stigma “is typically a social process, experienced or anticipated, characterized by exclusion, rejection, blame, or devaluation that results from experience or reasonable anticipation of an adverse social judgment about a person or group.” [42] (p. 13) Our sample included reflections about stigma in public health campaigns as well as in patient–doctor relations.

Stigma, as an external motivation to lose weight or even as a preventive measure, was discussed critically. Nath, for example, criticizes Callahans’s consequentialist standpoint^6^ that stigma may be justified in providing significant public health gains [44]. She also argues that adults with obesity are not blameworthy for their body weight and deserve non-stigmatizing treatment [45]. Abu-Odeh critically examines campaigns such as ‘cut the junk’ [46], which depicts a large man’s silhouette pouring crisps into his mouth [47]. Individuals targeted by stigmatizing health campaigns may face discrimination and a loss of social status due to being labelled as different. This includes the deprivation of opportunities and internalized self-stigmatization, both of which are unjust outcomes, as they harm the individual [47]. Furthermore, stigma can neither reduce nor prevent obesity [45]. In addition, stigmatizing public health campaigns are unfair since obesity is more prevalent among racial minorities and people with low SES. Weight stigma thus disproportionately targets already disadvantaged and marginalized groups [45, 47, 48]. 

Adults with obesity also experience stigma from healthcare professionals, driven by stereotypes that consider obesity a weakness of will [40] and may influence individual judgement, behaviour towards patients, and decision-making; [49] potentially limiting patients’ access to interventions and treatment [50].

#### Access to healthcare

##### Lower priority for patients who ‘caused’ their illness

Sharkey and Gillam argued that assigning lower priority to patients who have ‘caused’ their illness is morally problematic since it is not possible to assess each patient’s degree of responsibility. They further contended that such prioritization polices may decrease patient’s trust in healthcare professionals and requires healthcare professionals “to perform an inappropriate role judging or punishing patients” [38] (p. 663). Plus, a lower priority policy would only be fair if it applied to all ‘self-caused’ illnesses [38].

According to Eyal, denying healthcare for patients with ‘self-caused’ obesity also illustrates a turn to individualistic public health policy [51], which penalizes minorities and the economically worse off in a disproportionate way. Denying patients access to healthcare should therefore never be used as an incentive to become thinner. Refusing treatment may even contribute to comfort eating as a coping mechanism, thereby undermining rather than facilitating healthier eating habits [51]. 

##### Health gains from individualistic policies

Goldberg highlights that the degree to which a person can benefit from individualistic public health policies depends strongly on their monetary resources [52]. Compared with economically weak patients, richer patients are more likely to benefit from agentic interventions^7^. This was seen as a perpetual problem for interventions for adults with obesity. Coggon et al. for instance, showcase how personal, financial and time resources are required to enact individual agency but are less available to people living in less affluent circumstances. This implies that health inequalities increase even if the intervention successfully improves overall health [54]. 

##### Access to bariatric surgery

Hofmann emphasized that only a few patients in the US are offered bariatric surgery. This could be due to restrictive guidelines, which may reflect ingrained prejudices against adults with obesity or unbalanced advertisements. Within a private healthcare system such as that in the U.S. at that time, those who actually received surgery were not those with the highest BMI [40]. Saarni et al. state that unequal access to treatment is a problem of justice. Their conclusion for Finland constitutes that bariatric surgery should be publicly funded, as it should be seen as an effective treatment for a significant and disabling disease [39]. Craig et al. argue that bariatric surgery is cost-effective and cost-saving [50]. Denying the provision of bariatric service therefore “not only constitutes a breach of ethical justice but also seems unwise” [50] (p. 2081).

#### Prevention and food policy

The restriction of access to ‘unhealthy food and drink’ for people in the Supplement Nutrition Assistance Program (SNAP) was discussed contentiously. Such restriction may be justifiable as long as the participants have enough resources to pursue a life, they find valuable. Restricting sugary drink purchases with food stamps may seem disrespectful for SNAP users, but the policy could be justified if it leads to positive outcomes [55, 56]. Furthermore, the liberty of buying sugar-sweetened beverages (SSB) with food stamps may limit participants’ freedom, but such liberties were not deemed essential for well-being or self-determination [36].

In some papers, taxing SSB was evaluated as ethically sound if the food industry pays for the tax. Higher taxation of SSB may lead to a reduction in calories, and any revenue generated could be used for obesity prevention. Taxes can be unfair when applied to basic necessities such as food, but sugary soft drinks were not deemed a necessity [36]. Others dispute that taxing soft drinks is inherently regressive. Regardless of their impact on unhealthy eating, such taxes can be patronizing by limiting food choices for low-income people more than for wealthy people [57]. Telling lower income citizens what to buy with food stamps is yet another assault on the dignity of those who have already suffered most [58]. Additionally, economically disadvantaged consumers often still buy taxed foods but switch to lower-quality products or even cut back on healthier options to afford their favourite products [57].

#### Health as the ‘ultimate goal’

Thompson and Coveney highlight pleasure as a goal besides maintaining or improving health. Privileged people can freely choose their (costly) pastimes, which often excludes the less well-off, leaving them with (sometimes unhealthy) pleasures such as eating unhealthy food [59]. Others noted that not everyone sets health as their main goal or sees health as a necessary condition for a good life. Being healthy – or, more specifically, skinny – is itself not a unanimous ideal. Therefore, imposing personal costs on citizens with ‘risky’ lifestyles can lead to a form of moralizing that is incompatible with liberal values [37]. 

Wilkinson questions whether it would be unjust to reduce health inequity by improving the health of the worst off at the cost of other non-health goods^8^. Health is not so ethically paramount that it should take precedence over all other goods. If reducing inequality makes things worse for the people already struggling the most, then the gain in health equity might not be worth it – or it might show a mistaken focus on health as separate from well-being [60]. 

The social model of disability could help to make the case for a right to be fat, thus questioning health as the ultimate goal [57]. Governments must no longer advocate for weight loss or punish unhealthy behaviour but instead “provide for a holistic approach to health that recognises fat people as ‘equal members of a diverse humanity’” (57) (p. 168). Buchanan highlights that, for a healthy life, individuals must be able to make choices about the way they lead their lives, “including how fitness matters to them, relative to other priorities they may have” [61] (pp. 408/409).

### Notions of justice in the sample

To grasp how moral concerns about obesity are framed as matters of justice, it is crucial to consider the ways in which social justice was operationalized in the included texts: Some authors critically analysed lines of arguments that could potentially reinforce injustice, while others identified concrete, avoidable injustices affecting adults with obesity in public health, using theories of justice to claim for morally relevant changes.^9^ In the following, we also briefly illustrate how the concepts of (social) justice and equity were operationalized within our sample.

#### Arguments that risk reinforcing injustices

##### Desert-based approaches

In desert-based theory, “justice obtains when goods and evils are distributed according to desert” [62]. Several authors [45, 37, 61] critically examined whether the negative consequences associated with obesity (such as health outcomes or stigma) could be considered just and ‘deserved’ on the basis of ‘poor choices’. All rejected this view, pointing to the complex range of factors that influence body weight.

##### Paternalistic approaches

Some authors argued for a soft paternalism. This can be warranted if the true interest of an individual is clouded (i.e., ignorance, ingrained social behaviour, or temptations) [63]. If someone neglects their health due to flaws in their reasoning (i.e., preferring present pleasure to distant consequences), an unhealthy lifestyle is not truly voluntary. Soft paternalism can be a protective measure to prevent people from putting their health at risk [63]. However, such measures must be applied carefully to avoid reinforcing the very injustices they aim to address. Wilkinson argues that many preventive regulations – such as sugar taxes or restrictions on fast-food outlets – function paternalistically by limiting choices on the assumption that people, especially those who are socioeconomically disadvantaged, are choosing poorly [60]. Yet he cautions that there is little evidence that most disadvantaged individuals act against their own long-term preferences. Instead, their higher rates of obesity may stem from *structurally limited options*, not from flawed decision-making. When paternalistic policies reduce options for those who already have the fewest, they risk worsening their situation rather than improving it. In this way, such interventions can perpetuate or even intensify injustice, making them inequitable despite their public-health intentions [60]. 

##### Libertarianism

A libertarian approach aims for “a maximum of liberty and property resulting from the exercise of liberty rights and participation in fair free-market exchanges” [14] (p. 271). In one case from our sample, this was contrasted with utilitarianism to debate health insurance for adults with obesity [64]. An overemphasized individual right may lead to heightened personal responsibility and less focus on the impact that personal decisions have on others; it further runs the risk of ignoring the impact of structural and societal inequities [64]. By also acknowledging such inequities, the authors conclude that libertarian and utilitarian approaches (which serve the greater good by improving public health, reducing costs, and fostering healthier future generations) need to be incorporated to decide upon health insurance coverage for adults with obesity.

##### Utilitarianism

Utilitarianism was used mainly to discuss stigmatizing campaigns, campaigns that use weight loss as incentives [51] or policies that restrict certain foods and drinks for participants in nutritional support programs [55, 56]. Critics hold that pure utilitarian accounts cannot reduce obesity and run the risk of disproportionately targeting racial minorities or people of low SES. This is yet another assault on the dignity of those who have already suffered most [58]. 

##### The operationalization of the term (social) justice and equity in our sample

As mentioned in the background, there is a need to differentiate between health equity and social justice. Social justice comprises multiple societal dimensions that extend beyond the health sector, whereas health equity risks focusing on the differences in health outcomes easiest to address, even at the cost of inequalities in other social spheres [9]. A clear distinction between the terms ‘equity’ and ‘justice’ *did not* permeate our sample. The terms ‘equity’ (in some text, even ‘equality’) and ‘justice’ were commonly used interchangeable. Buchanan was the only author explicitly critiquing this undifferentiated use of terms [61].

#### Theories of justice as a foundation for public health (ethics)

Detecting health injustices and developing health policies to avoid future health injustices are two core tasks of public health. In our sample, theories of justice were used for this exact purpose. Three theoretical approaches (fairness, capabilities, and relational egalitarianism)^10^ were used to identify health injustices and how future policies should be shaped to avoid health injustices. In the next part of the results, we first outline the relevant theories and then examine how they were employed across our text corpus.

##### Fairness

Based on John Rawls [65], this approach was further developed by Norman Daniels [66]. It emphasizes health needs as a central aspect of distributive justice, arguing that justice requires fair equality of opportunity, which includes access to healthcare. According to Daniels, good health is crucial for individuals to participate in society, and a just society must reduce health disparities and provide access to essential health services. When allocating scarce health resources, Daniels suggests that the process of policy making should be publicly accessible, transparent, justifiable, and revisable [66] (pp.118/119).

This approach was referred to multiple times in our sample. Obesity often impairs “normal opportunity range” (66) (p. 43), leads to lower life expectancy/quality and, therefore, to unequal opportunities [67]. Consequently, treating obese patients provides them with options for taking up opportunities. However, Schermer points out that this concept overlooks how citizens become obese. She concludes that the society is not obliged to provide treatment unless people have a monogenetic disposition for obesity [67]. 

Baumrucker et al.’s ethics roundtable evaluates a US case with a patient who has access to health insurance but chooses to give up on it to pay for his daughter’s education. Due to his severe obesity and Pickwinian syndrome, however, the patient needs healthcare [68]. One participant presents the ‘fair opportunity rule’. The rule states that no one should be denied social benefits because of other disadvantages they face. Applying this rule can counteract the unfortunate effects of life’s lottery^11^: if a patient is born with genetic properties that lead to social disadvantages, healthcare costs should be covered. However, in this case, the patient chooses not to take up health insurance. On the basis of this case, the participant proposes a ‘decent minimum standard’ consisting of two tiers of health care coverage. Tier 1 covers basic and catastrophic health needs met by everyone, with or without health insurance coverage. Tier 2 covers other health care needs and desires depending on whether one has satisfied the criteria/material principles set for allowing such coverage.

Morain used Rawls’ “liberal principle of legitimacy”^12^ to review two public health strategies. The first strategy addresses laws mandating that restaurants display nutritional information on their menus. The second looks into laws mandating that individuals participate in daily exercise. Morain maintains that the former poses no restriction on basic liberties, whereas the latter violates personal integrity without serving another basic liberty and is therefore unacceptable [69]. 

##### Capabilities

When many options are foreclosed or limited by the effects of poverty, inferior education, racism, or disproportionate exposure to environmental toxins, the primary and principal concern of justice should be what individuals are actually able to achieve. In the capabilities approach (CA), ‘functionings’ are the actual “doing and beings” [15] (p. 31) a person can achieve, such as being healthy, educated or employed. ‘Capabilities’ refer to the real opportunities available to attain these “doings and beings” [15] (p. 31). Measuring justice depends on the degree to which people are capable of achieving valuable functionings under certain conditions. In this way, CA brings attention to social conditions that impair an individual’s motivation to act reflectively.

Buchanan proposes that by incorporating the CA into policymaking, people should be able to pursue a broader range of respect-worthy activities [61]. Lewis states that the CA is particularly suited to evaluate the built environment for physical activity in the context of obesity and to broaden the scope beyond the urban white middle class. Previous research has left out marginalized groups that face major barriers to using their environment for physical activity, such as unsafe neighbourhoods. The CA helps reveal and assess these limits, showing how lacking one resource can prevent some from using another, even if it is widely available [70]. 

##### Relational egalitarianism

Iris Young’s account of relational egalitarianism and political responsibility emphasizes social structures as dominant drivers of injustice and advocates for changes that empower disadvantaged communities and reduce systemic inequalities. These factors determine the position in which individuals find themselves in relation to others. Their social position shapes which opportunities are available or constrained. These conditions cannot be changed by individual action but rather by collective action [71]. Tulatz uses this approach to highlight the injustice adults with obesity experience and to shed light on three social aspects of obesity prevention. First, small local civic initiatives can serve as examples of alternative approaches. Second, all actors responsible for driving change should be involved. Third, discussion should be open about the stakes when obesity is treated as a political issue, encouraging disadvantaged groups to ally and express their needs [72]. 

Tempels et al. highlight how food-related public health problems are connected to the food industry and can be understood, drawing on the work of Young, as structural injustice. Selling products that exceed daily caloric needs, stalling policies, creating food deserts^13^ or advertising, especially for people with a lower SES, reinforce social norms and systems that further disadvantage marginalized groups. The food industry is responsible for the resulting structural injustices and needs to address them. However, they reported that clear and concrete parameters for attributing responsibility are lacking [74]. 

## Discussion

### Summarizing the results

Although social justice is a leading aim in public health interventions and policymaking, ethical reflection is still emerging in many fields. The most discussed issues were responsibility, stigma, obesity as a disease, preventive measures and health as the ultimate goal. With respect to theories of justice, we discovered an array of theoretical approaches that were used for different purposes.

Most texts in the sample highlighted that obesity *is* a matter of social justice. The majority of authors have analysed why current interventions, policies, or ideas are unjust and how different lines of argument risk further injustices. However, only a few authors also used specific theories of justice to back their arguments and formulated initial efforts for future policymaking.

### Dimensions of social justice for adults with high body weight

Only one text in our sample provided an initial holistic definition of social justice for adults with high body weight (AHBW)^14^: Russel-Mayhew and Grace posit that the key dimensions of social justice are resource distribution, physical and psychological security as well as power relations [75]. This approach can offer insights into how social categories, relationships, and power dynamics shape people’s connection to their bodies.

We took this definition as an impulse to develop a multisited notion of social justice for AHBW by incorporating the main ethical concerns identified in this review. While a comprehensive analysis of theories of justice was beyond the scope of this review, we nonetheless briefly referenced relevant theories of social justice to illustrate how different approaches could support various aspects of social justice for AHBW. In doing so, we acknowledge that there is not *one* theory of justice that can be used for every practical need (such as resource allocation, clinical interactions, assigning responsibility, or health policy making). Rather, different purposes and issues need varied approaches with different metrics of social justice involved in at least three dimensions.

- Healthcare: This dimension points to adequate and equal access to medicine and prevention. Medical encounters should not be imposed and in a manner free from discrimination and stigma [76, 77]. Practical tools for allocating resources, for revising guidelines and setting priorities could be developed using the *fairness approach* [66]. Following this, social justice requires equal opportunity for AHBW, which depends on access to healthcare. Ensuring this access is the responsibility of the healthcare system [66]. Achieving this in practice requires the systematic removal of financial, geographical, and sociocultural barriers; the protection of patient’s rights to informed and voluntary care; and the implementation of training and policies that minimize bias among healthcare professionals and reduce structural discrimination. It is also crucial to recognize that AHBW are a diverse group with varying healthcare needs, unique histories and emotions. A personalized approach is essential for improving health outcomes and quality of life [78, 79]. Another important factor is surely also the structure of healthcare systems, as roles, responsibilities, and requirements can differ greatly between models such as private insurance and state-funded care.
- Society: This dimension highlights the need for a society that recognizes the complex, multifactorial causes of high body weight without resorting to stigma or the notion of personal blame. This emphasizes that health inequalities shaped by the SDH require a nuanced ethical discourse to challenge prejudice and communicate complexity in public discourse, education, and everyday life. *Relational egalitarianism* [71] offers a framework by focusing on dismantling societal structures – such as bias, discrimination, and hierarchy – that deny individuals’ equal moral respect. In practice, this can be achieved through inclusive media representation [80, 81], consistent use of weight-neutral language in public and professional communication [82, 83], and initiatives that involve all actors responsible for determining what is needed to generate change. These efforts can help foster more social relations and reduce weight-based stigma. In addition, initiatives are needed to support health scholars and health professionals in reflecting their own attitudes, language, and behaviour to contribute to building a more just, respectful, and stigma-free society [84].^15^
- Politics: It is essential to recognize the political dimension in relation to the SDH of high body weight. Just and effective policymaking must consider the complex interconnections among the social, economic, and environmental factors that influence health [86]. The *capabilities approach* helps identify what individuals can actually achieve, highlighting gaps between potential and realized functionings. Moreover, tackling injustices related to high body weight at the political level requires attention to the full complexity of public health decision-making while safeguarding democratic processes [87]. Therefore, Nancy Fraser’s third *scale of justice* – representation – is an important reference point: “Misrepresentation occurs when political boundaries and/or decision rules function wrongly to deny some people the possibility of participating on a par with others in social interaction – including, but not only, in political arenas.” [88] (p. 18) To remedy such injustices, Fraser emphasizes reforming decision rules, expanding channels for marginalized voices, and removing structural barriers to meaningful participation, ensuring all individuals can engage as peers in social and political life [88]. In practice, participatory approaches can operationalize these principles: by involving AHBW in policy development and implementation, decision-makers can design solutions that are both theoretically sound and practically effective in addressing the complex realities this populations faces [89, 90].

Looking at one particular case from different theoretical perspectives, it becomes apparent that there is no ultimate theory of justice when reflecting on the situation of AHBW. Surely, a philosophical debate about these theories is important; however, our analysis shows that there is a wide array of possible theoretical approaches that solidify our understanding of justice. This is especially important when we think about translating ethical considerations about justice into practice to inform policy makers or health care professionals and students. Theories of justice can be a powerful tool for advancing public health aims for social justice, particularly when they help identify existing injustices and guide future framework development. Importantly, although some strategies exist, such as stigma-free clinical guidelines or inclusive media recommendations, further nuanced interventions are needed to address the full spectrum of social justice for AHBW.

### Strengths and limitations

Our paper provides a systematic review of social justice for AHBW, including the use of theoretical approaches. However, we also want to highlight the limitations of this paper:

Methodological limitations: Our search terms were only applied to title, abstract and keywords. As a result, ethical discussions about social justice appearing solely in the full text were not captured in this systematic review, indicating that additional relevant texts likely exist.

Potential biases: Additionally, our inclusion criterion to include only English texts may lead to some texts being excluded as well as a biased western point of view on ethical issues.

Gaps in reviewed literature: Our latest text included in this sample was from 2021 (although our database search was in 2024), so recent developments in weight loss drugs were not included in this review. These new drugs are nonetheless also an essential topic of discussion about social justice for AHBW.

## Conclusion

On the basis of this systematic review, we develop an integrated, multidimensional understanding of social justice for AHBW. This highlights the practical use of theories of justice beyond mere models and abstracted practical implications that should follow. We show that an array of ethical issues concerning justice and high body weight are currently discussed. The aim of establishing high body weight as an issue of social justice and the aim for health equity permeated our sample, whereas the practice of supporting such claims through a concrete understanding of justice was just emerging. Our review nonetheless showed that specific understandings of justice are essential to go beyond distribution and incorporate wider perspectives as well as societal dimensions. Future translational efforts are needed to incorporate abstract models of theories of justice into practical tools for healthcare staff and policy makers.

## Supplementary Information

Below is the link to the electronic supplementary material.

Supplementary Material 1

Supplementary Material 2

## Data Availability

No datasets were generated or analysed during the current study.

## Footnotes

1 The term ‘obesity’ is used in the early sections of this paper to reflect the language of the literature review and to align with the search strategy. In the discussion, we shift to more weight-neutral terms – such as ‘adults with high body weight’ – to align with an inclusive discourse.

2 Here moral justification does not depend in a single foundational rule but instead emerges from the coherence and mutual support among multiple beliefs and principles [14] (p.44).

3 In the *well-being approach*, Powers and Faden [11] identify six essential dimensions of well-being: health, personal security, reasoning, respect, attachment, and self-determination, which should be distributed to ensure a sufficient level of well-being. Their account also highlights what they describe as the twin moral impulses that animate public health: the commitment to improving human well-being by strengthening health and the other essential dimensions of well-being, and the obligation to do so with particular attention to those who are most disadvantaged. [11] The *capabilities approach* defines ‘functionings’ as the actual “doing and beings” (15) (p.31) a person can achieve, such as being healthy, educated or in secure employment. ‘Capabilities’ are the possibilities people actually have to achieve these “doings and beings. In other words, measuring justice depends on the degree to which people are capable of achieving valuable functionings under certain conditions.

4 For example, the risk of cardiometabolic multimorbidity is five times higher in people with grade I obesity and more than ten times higher for people with severe obesity [17].

5 For example: [34, 35].

6 In consequentialist ethical theories, the moral rightness of an action depends solely in its consequences. Actions are judged as good or bad based on their outcome, not in principles, rules, or duties [43].

7 Agentic interventions emphasize offering advice, guidance, and encouragement, but they depend largely on individuals’ ability and motivation to engage with this support. To benefit, recipients must draw on their personal resources. [53]

8 Non-health goods could be income, education or (social) freedom.

9 Notably, a coherentist framework [14] was used quite frequently as an analytical tool to evaluate case studies, which predominantly focused on distributive issues, such as allocation of healthcare resources, insurance coverage, or access to bariatric surgery.

10 Whilst many authors highlighted the important work of Powers and Faden, none applied their well-being approach as a theory of justice.

11 Referring to John Rawls [65], who differentiates the natural lottery (genetics) and social lottery (assets, property, schools, and government organizations).

12 Rawls states that political power is legitimate when exercised according to a constitution that all reasonable and rational citizens can endorse. With that the aim is also to secure a zone of non-interference over choices and focuses on a defined set of basic liberties. Those basic liberties have the absolute priority over social and economic efficiency considerations. Furthermore, they should only be limited to protect other basic liberties [65].

13 Areas with limited access to grocery shops that supply to ‘healthy’ and affordable food [73].

14 To highlight a more inclusive and weight-neutral discourse, we switch to the term “adults with high body weight” in the discussion.

15 Rebecca Puhl for example, provides a comprehensive overview about which (interdisciplinary) efforts – particularly regarding stigma towards AHBW – should be undertaken [85].
