# Supplementary material for: Social justice for adults with high body weight: a systematic review
Source: Int J Equity Health. 2026 Feb 21;25:56. doi: 10.1186/s12939-026-02792-4 (PMC12933919; doi:10.1186/s12939-026-02792-4)
Supplement: Supplementary file 1 — Supplementary Material 1 [file 12939_2026_2792_MOESM1_ESM.docx]

Supplementary Material 2: References included texts in review

1. Schermer M. Genomics, obesity and enhancement: moral issues regarding aesthetics and health. Genomics, Society and Policy 2008; 4(2):36–52.

2. Hofmann B. Stuck in the Middle: The Many Moral Challenges with Bariatric Surgery. The American Journal of Bioethics 2010; 10(12):3–11.

3. Sharkey K, Gillam L. Should patients with self-inflicted illness receive lower priority in access to healthcare resources? Mapping out the debate. Journal of Medical Ethics 2010; 36(11):661–5.

4. Goldberg DS. What Kind of People: Obesity Stigma and Inequities. The American Journal of Medicine 2011; 124(8):788.

5. Saarni, Samuli, I, Anttila H, Saarni, Suoma, E., Mustajoki P, Koivukangas V, Ikonen, Tuija, S. et al. Ethical Issues of Obesity Surgery — a Health Technology Assessment. Obesity Surgery 2011; 21(9):1469–76.

6. Baumrucker SJ, Stolick M, Mingle P, Oertli KM, Vandekieft G. The Principle of Distributive Justice. American Journal of Hospice & Palliative Medicine 2012; 29(2):151–6.

7. Greer AG, Chapman C, Butler Ryckeley J. Moral Hazard or Morality for Health: An Ethical Debate on Insurance Coverage for the Obese. Bariatric Surgical Practice and Patient Care 2012; 7(1):36–41.

8. Lewis F. Auditing Capability and Active Living in the Built Environment. Journal of Human Development and Capabilites 2012; 13(2):295–315.

9. Barnhill A, King KF. Ethical Agreement and Disagreement about Obesity Prevention Policy in the United States. International Journal of Health Policy and Management 2013; 1(2):117–20.

10. Barnhill A, King KF. Evaluating Equity Critiques in Food Policy: The Case of Sugar-Sweetened Beverages. Journal of Law, Medicine & Ethics 2013; 41(1):301–9.

11. Buchanan D. Ethical Standards to Guide the Development of Obesity Policies and Programs: Comment on “Ethical Agreement and Disagreement about Obesity Prevention Policy in the United States”. International Journal of Health Policy and Management 2013; 1(4):313–5.

12. Eyal N. Denial of Treatment to Obese Patients—the Wrong Policy on Personal Responsibility for Health. International Journal of Health Policy and Management 2013; 1(2):107–10.

13. Gallagher S M. Meaningful Ethical Tools for the Quality of Life Debate. Bariatric Surgical Practice and Patient Care 2013; 8(2):53-57.

14. Goldberg DS. The Errors of Individualistic Public Health Interventions: Denial of Treatment to Obese Persons: Comment on “Denial of Treatment to Obese Patients—the Wrong Policy on Personal Responsibility for Health”. International Journal of Health Policy and Management 2013; 1(3):237–8.

15. Abu-Odeh D. Fat Stigma and Pubic Health: A Theoretical Framework and Ethical Analysis. Kennedy Institute of Ethics Journal 2014; 24(3):247–65.

16. Kass N, Hecht K, Paul A, Birnbach K. Ethics and Obesity Prevention: Ethical Consideration in 3 Aprroaches to Reducing Consumption of Sugar-Sweetened Beverages. American Journal of Public Health 2014; 104(5):787–95.

17. Nielsen MEJ, Andersen MM. Should we Hold the Obese Responsible? Some Key Issues. Cambridge Quarterly of Healthcare Ethics 2014; 23(4):1–9.

18. Buchanan DR. Promoting Justice and Autonomy in Public Policies to Reduce the Health Consequences of Obesity. Kennedy Institute of Ethics Journal 2015; 25(4):395–417.

19. Kniess J. Obesity, paternalism and fairness. J Med Ethics 2015; 41(11):889–92.

20. Morain S. Evaluating the Legitimacy of Contemporary Legal Strategies for Obesity. Kennedy Institute of Ethics Journal 2015; 25(4):369–93.

21. Russel-Mayhew S, Grace AD. A Call for Social Justice and Best Practices for the Integrated Prevention of Eating Disorders and Obesity. Eating disorders 2016; 24(1):54–62.

22. Schneider PL. Li, Z. Ethical Challenges in the Care of the Inpatient with Morbid Obesity. Narrative Inquiry in Bioethics 2016; 6 (2): 143-152.

23. Thompson L, Coveney J. Human vulnerabilities, transgression and pleasure. Critical Public Health 2017; 28(1):118–28.

24. Craig H, Le Roux, Carel, Keogh, Fiona, Finucane FM. How Ethical Is Our Current Delivery of Care to Patients with Servere and Complicated Obesity? Obesity Surgery 2018; 28(7):2078–82.

25. Tulatz K. Obesity, political responsibility, and the politics of needs. Med Health Care and Philos 2019; 22(2):305–15.

26. Wilkinson TM. Obesity, Equity and Choice. Journal of Medical Ethics 2019; 45(5):323–8.

27. Nath R. The Injustice of Fat Stigma. Bioethics 2019; 33:577–90.

28. Freeman L. A Matter of Justice: "Fat" is Not Necessarily a Bad Word. Hastings Center Report 2020; 50(5):11–6.

29. Tempels T, Blok V, Verweij M. Injustice in Food-Related Public Health Problems: A Matter of Corporate Responsibility. Bus. Ethics Q. 2020; 30(3):388–413.

30. Coggon J, Adams J. ‘Let them choose not to eat cake…’: Public health ethics, effectiveness and equity in government obesity strategy. Future Healthcare Journal 2021; 8(1):49–52.

31. McPhail D, Orsini M. Fat Acceptance as Social Justice. CMAJ 2021; 193(35):E1398-E1399.

32. Kanagasingam D, Norman M, Hurd L. Illuminating the Ethical Tensions in the Obesity Canada Website: A Transdisciplinary Social Justice Perspective. Journal of Critical Realism 2021; 20(5):474–90.

33. Schorb F. Crossroad between the right to health and the right to be fat. Fat Studies 2021; 10(2):160–71.
