# Supplementary material for: Social justice for adults with high body weight: a systematic review
Source: Int J Equity Health. 2026 Feb 21;25:56. doi: 10.1186/s12939-026-02792-4 (PMC12933919; doi:10.1186/s12939-026-02792-4)
Supplement: Supplementary file 2 — Supplementary Material 2 [file 12939_2026_2792_MOESM2_ESM.docx]

Included articles and characteristics:

| **Article Reference** | **Author(s)** | **Title** | **Journal** | **Authors Background** | **Discussed Aspect of Obesity** |
| --- | --- | --- | --- | --- | --- |
| [1] | Schermer | Genomics, Obesity and Enhancement: Moral Issues Regarding Aesthetics and Health | Genomics, Society and Policy | Medical Ethics | Genomic enhancement |
| [2] | Hoffmann | Stuck in the Middle: The Many Moral Challenges with Bariatric Surgery | The American Journal of Bioethics | Medical Ethics | Bariatric surgery |
| [3] | Sharkey & Gillam | Should Patients with Self-inflicted Illness Receive Lower Priority in Access to Healthcare Resources? Mapping out the Debate | Journal of Medical Ethics | Interdisciplinary Team: Philosophy & Ethics | Lower priority in healthcare access for people with self-induced illness |
| [4] | Goldberg | What Kind of People: Obesity Stigma and Inequities | The American Journal of Medicine | Bioethics | Stigma |
| [5] | Saarni et al. | Ethical Issues of Obesity Surgery - a Health Technology Assessment | Obesity Surgery | Interdisciplinary Team: Psychiatrist, Health Technology Assessment (HTA) & Medicine | Bariatric surgery |
| [6] | Baumrucker et al. | The Principle of Distributive Justice | American Journal of Hospice & Palliative Medicine | Interdisciplinary Team:  Family Medicine, Nursing,  Law, Palliative Care Physician & Clinical Ethics | Case discussion: access to healthcare |
| [7] | Greer et al. | Moral Hazard or Morality for Health: An Ethical Debate on Insurance Coverage for the Obese | Bariatric Nursing and Surgical Patient Care | Interdisciplinary Team: Bioethics & Medicine | Health-insurance for PwO |
| [8] | Lewis | Auditing Capability and Active Living in the Built Environment | Journal of Human Development and Capabilities | Urban Planning | Built-Environment Audits |
| [9] | Barnhill & King | Ethical Agreement and Disagreement About Obesity Prevention Policy in the United States | International Journal of Health Policy and Management | Interdisciplinary Team: Medical Ethics & Public Health | Obesity prevention policy (tax on unhealthy food and ban of soda) |
| [10] | Barnhill & King | Evaluating Equity Critiques in Food Policy: The Case of Sugar-Sweetened Beverages | Journal of Law, Medicine & Ethics | Interdisciplinary Team: Medical Ethics & Public Health | Restriction of sweetened beverages for SNAP-participants |
| [11] | Buchanan | Ethical Standards to Guide the Development of Obesity Policies and Programs Comment on “Ethical Agreement and Disagreement about Obesity Prevention Policy in the United States” | International Journal of Health Policy and Management | Public Health Ethics | Obesity prevention - Comment on Barnhill & King |
| [12] | Eyal | Denial of Treatment to Obese Patients—the Wrong Policy on Personal Responsibility for Health | International Journal of Health Policy and Management | Bioethics | Access to treatment |
| [13] | Gallagher | Meaningful Ethical Tools for the Quality of Life Debate | Bariatric Surgery Practice and Patient Care | Medical Ethics | Distribution of resources, thics of Care, interpersonal decision making |
| [14] | Goldberg | The Errors of Individualistic Public Health Interventions: Denial of Treatment to Obese Persons Comment on “Denial of Treatment to Obese Patients - the Wrong Policy on Personal Responsibility for Health” | International Journal of Health Policy and Management | Bioethics | Denying obese patients’ treatment |
| [15] | Abu-Odeh | Fat Stigma and Public Health: A Theoretical Framework and Ethical Analysis | Kennedy Institute of Ethics Journal | Bioethics | Public health interventions that use stigma causing harm, loss of agency and injustice |
| [16] | Kass et al. | Ethics and Obesity Prevention: Ethical Considerations in 3 Approaches to Reducing Consumption of Sugar-Sweetened Beverages | American Journal of Public Health | Interdisciplinary Team: Public Health, Bioethics & Food Policy/Advocacy | Public health intervention to reduce SSB consumption |
| [17] | Nielsen & Andersen | Should we Hold the Obese Responsible? Some Key Issues | Cambridge Quarterly of Healthcare Ethics | Interdisciplinary Team: Philosophy & Public Health | Denying obese patients’ treatment |
| [18] | Buchanan | Promoting Justice and Autonomy in Public Policies to Reduce the Health Consequences of Obesity | Kennedy Institute of Ethics Journal | Public Health Ethics | Obesity policies |
| [19] | Kniess | Obesity, Paternalism and Fairness | Journal of Medical Ethics | Political Philosophy | Restricting policies for obese/food lovers |
| [20] | Morain | Evaluating the Legitimacy of Contemporary Legal Strategies for Obesity | Kennedy Institute of Ethics Journal | Bioethics | Obesity prevention policy (menu labelling and mandatory exercise) |
| [21] | Russell-Mayhew & Grace | A Call for Social Justice and Best Practices for the Integrated Prevention of Eating Disorders and Obesity | Eating Disorders | Psychology | Prevention of weight-related issues (Eating-Disorder and Obesity) |
| [22] | Schneider & Li | Ethical Challenges in the Care of the Inpatient with Morbid Obesity | Narrative Inquiry in Bioethics | Medicine | Inpatient care for obese patients |
| [23] | Thompson & Coveney | Human Vulnerabilities, Transgression and Pleasure | Critical Public Health | Social Science, Nursing, & Health Science | Experience pleasure in food |
| [24] | Craig et al. | How Ethical Is Our Current Delivery of Care to Patients with Severe and Complicated Obesity? | Obesity Surgery | Interdisciplinary Team: Medicine, Mental Health, Economic, & Social Research | Bariatric surgery |
| [25] | Tulatz | Obesity, Political Responsibility, and the Politics of Needs | Medicine, Health Care and Philosophy | Philosophy | Obesity prevention |
| [26] | Wilkinson | Obesity, Equity and Choice | Journal of Medical Ethics | Politics & International Relations | Obesity prevention |
| [27] | Nath | The Injustice of Fat Stigma | Bioethics | Philosophy | Justice and stigma |
| [28] | Freeman | A Matter of Justice: “Fat” Is Not Necessarily a Bad Word | Hastings Center Report | Philosophy | Stigma, healthcare provision |
| [29] | Tempels et al. | Injustice in Food-Related Public Health Problems: A Matter of Corporate Responsibility | Business Ethics Quarterly | Political Theory & Philosophy | Corporate responsibility for food-related injustice |
| [30] | Coggon & Adams | ‘Let them choose not to eat cake…’: Public Health Ethics, Effectiveness and Equity in Government Obesity Strategy | Future Healthcare Journal | Interdisciplinary Team: Law & Dietary Public Health | Obesity prevention |
| [31] | McPhail & Orsini | Fat acceptance as social justice | Canadian Medical Association Journal | Community Health  Sciences & Feminist and Gender Studies | Confronting fat stigma |
| [32] | Kanagasingam et al. | Illuminating the ethical tensions in the obesity Canada website: a transdisciplinary social justice perspective | Journal of Critical Realism | Socio-Cultural Studies | Application of social justice lens onto the obesity Canada website |
| [33] | Schorb | Crossroad Between the Right to Health and the Right to be Fat | Fat Studies | Sociology | Right to be fat vs. right to be healthy |
